# Giant Primary Cutaneous Myoepithelial Carcinoma of the Left Thigh With Inguinal and Pelvic Lymph Node Metastases

**DOI:** 10.7759/cureus.68571

**Published:** 2024-09-03

**Authors:** Ayaka Obata, Hikaru Kawahara, Hitomi Sugino, Yoko Amagata, Natsuko Saito-Sasaki, Etsuko Okada, Yu Sawada

**Affiliations:** 1 Dermatology, University of Occupational and Environmental Health, Kitakyushu, JPN

**Keywords:** surgical resection, chemotherapy, case report, skin cancer, myoepithelial carcinoma

## Abstract

Myoepithelial carcinoma is an exceedingly rare malignancy, particularly when originating from the skin. It frequently arises from malignant transformations of pleomorphic adenomas in various locations such as the parotid gland, breast, soft tissues, and lungs. Primary cutaneous myoepithelial carcinoma is exceptionally rare, often leading to delayed diagnosis. We report a case of giant primary cutaneous myoepithelial carcinoma of the left thigh, initially misdiagnosed as squamous cell carcinoma (SCC). The patient, a 64-year-old male, presented with a rapidly enlarging, ulcerated, and necrotic skin lesion. The initial presentation mimicked SCC. Due to the large tumor size and anemia caused by the tumor, the patient underwent a reduced-dose chemotherapy regimen (cytarabine plus aclarubicin chemotherapy) to shrink the tumor, enabling successful local surgical resection. Post-surgery, the patient received radiotherapy and tegafur gimeracil oteracil potassium, resulting in disease control without progression for two years. This case highlights the diagnostic challenges of myoepithelial carcinoma, which can mimic SCC among numerous other tumors. Accurate diagnosis relies on immunohistochemical staining and careful pathological evaluation. The case underscores the importance of considering myoepithelial carcinoma in the differential diagnosis of ulcerative tumors.

## Introduction

Myoepithelial carcinoma is an exceedingly rare malignancy, with a primary cutaneous origin even rarer [1,2]. This tumor is most commonly reported to arise from malignant transformations of pleomorphic adenomas, typically found in the parotid gland, breast, soft tissues, and lungs [1]. Due to the rarity of primary cutaneous myoepithelial carcinoma, physicians often struggle to make timely diagnoses, leading to delays in treatment [3,4]. Furthermore, there might be a delay due to the pathological diagnosis process, which requires additional time for sample collection, preparation, and analysis.

In this report, we present a case of a giant primary cutaneous myoepithelial carcinoma of the left thigh, a site that is highly unusual for this type of tumor. The carcinoma initially presented with clinical features that closely mimicked squamous cell carcinoma (SCC), basal cell carcinoma, Merkel cell carcinoma, and porocarcinoma, including a scaly, erythematous surface with ulceration. These features, along with the atypical location of the tumor, contributed to the initial misdiagnosis [5].

Myoepithelial carcinoma typically exhibits a combination of epithelial and myoepithelial cell characteristics, contributing to its complex histopathological profile [6]. The tumor cells often show a wide range of morphologies, including spindle, plasmacytoid, and epithelioid forms. Immunohistochemically, myoepithelial carcinoma cells express a variety of markers such as α-smooth muscle actin (α-SMA), muscle actin (designated HHF35), and calponin [7], reflecting the dual epithelial and myoepithelial differentiation. The presence of these markers, particularly the co-expression of epithelial and myoepithelial markers, is crucial for accurate diagnosis.

This case underscores the importance of considering myoepithelial carcinoma in the differential diagnosis of skin tumors with unusual presentations. Early recognition and accurate diagnosis are critical for optimal patient outcomes, as these tumors can be highly aggressive and may require a combination of surgical, radiological, and systemic treatments to achieve the best prognosis.

## Case presentation

**Figure 1:**
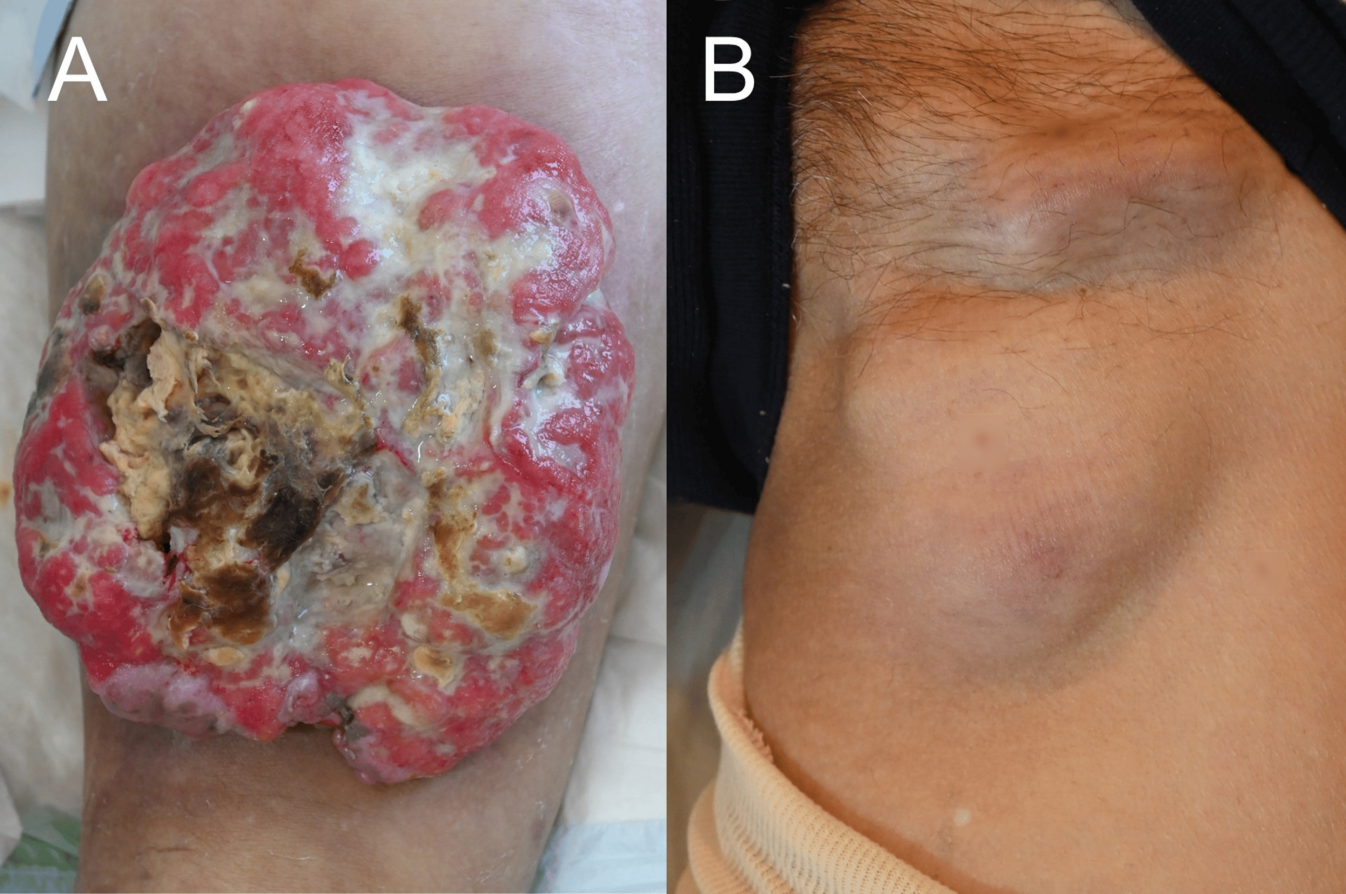
Clinical presentation of the tumor (A) A clinical image of the 20×20 cm pedunculated, red, raised lesion on the posterior aspect of the left thigh. The lesion showed an ulcerated and eroded surface with black and yellow necrosis. (B) A clinical image of the marked lymphadenopathy in the left inguinal region.

A 64-year-old male patient had noticed a small nodule in his left thigh 15 years prior, and the nodule began to enlarge rapidly in the previous year. There were no risks of trauma, either in his personal life or at work. He was referred to our department for the evaluation of his skin tumor. At the initial physical examination, a 20×20 cm large, pedunculated, red, raised lesion with poor mobility was observed on the posterior aspect of the left thigh. The lesion was ulcerated and eroded. The tumor presented large areas of necrosis and slough as well as being prone to bleeding (Figure 1A). Additionally, there was marked lymphadenopathy in the left inguinal region (Figure 1B), and clinically, SCC was initially suspected, along with other possibilities such as basal cell carcinoma, Merkel cell carcinoma, and porocarcinoma. The lymphadenopathy presented with strong adhesion to the surrounding tissues, resulting in limited mobility. The nodule was noted to be firm and hard upon palpation. Notably, there was an absence of tenderness or pain associated with the lymph node.

**Figure 2:**
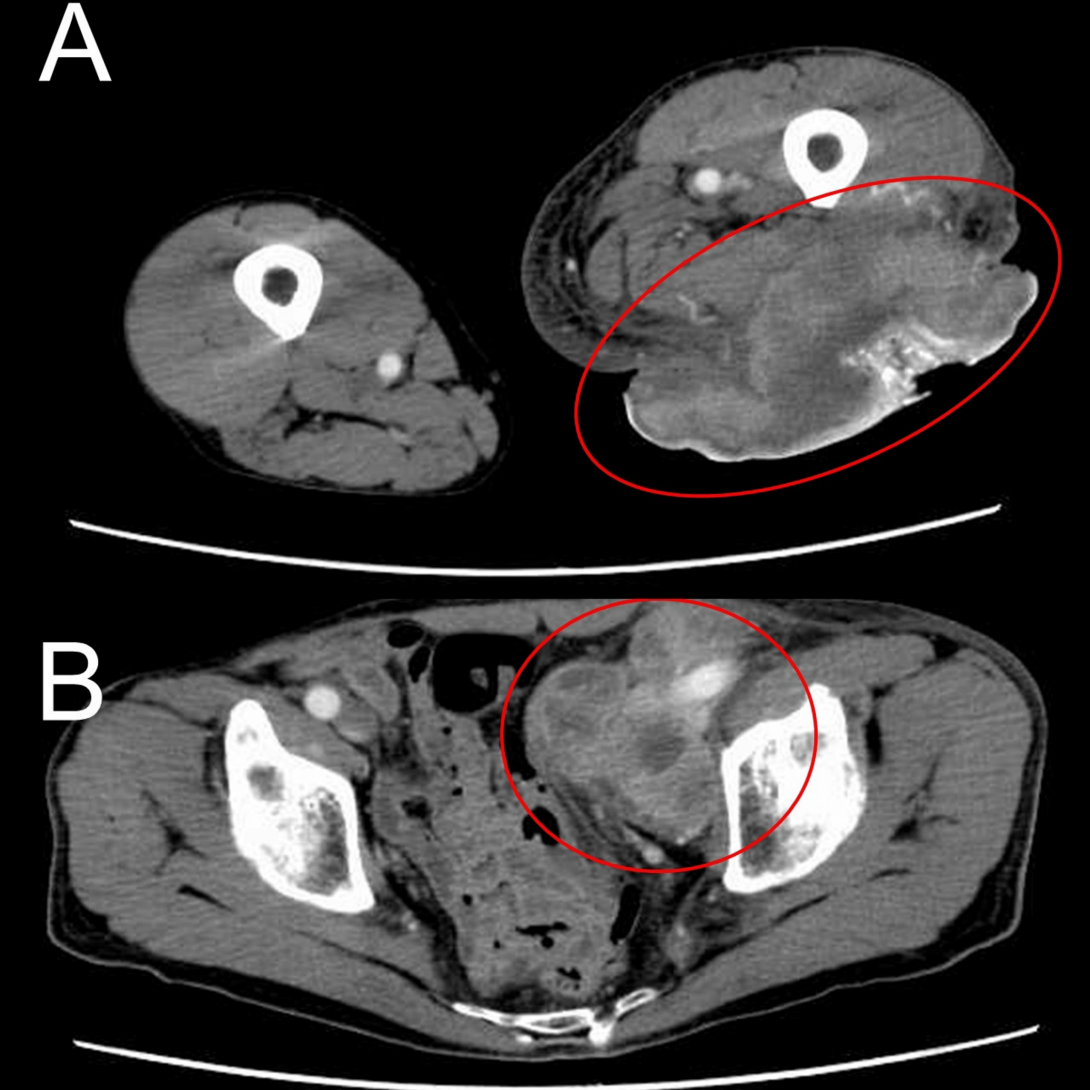
CT imaging findings (A) A contrast-enhanced CT image showing the large mass in the posterior left thigh, with areas of enhancement and necrosis. The tumor and necrotic areas are indicated. (B) A CT image showing multiple enlarged lymph nodes from the left external iliac to the para-aortic regions.

Contrast-enhanced CT at the initial visit showed a large mass in the posterior left thigh with areas of enhancement and poor contrast, suggestive of necrosis (Figure 2A). Multiple enlarged lymph nodes were detected from the left external iliac to the para-aortic regions, in contact with the left external iliac artery and femoral artery (Figure 2B). Based on the initial skin biopsy taken from the tumor, the diagnosis was primary cutaneous myoepithelial carcinoma. An imagistic evaluation revealed no metastasis to other organs; however, there was bleeding from the tumor, leading to anemia (hemoglobin 7.3 g/dL), which required regular blood transfusions.

Because it was difficult to achieve complete resection due to the large tumor size, the patient received the first cycle of CA (Cytarabine plus Aclarubicin) chemotherapy at full dose, resulting in grade 3 neutropenia. The second cycle of CA chemotherapy was administered at 80% of the cisplatin dose and 67% of the adriamycin dose to prevent severe neutropenia. The reduced-dose chemotherapy was administered to shrink the tumor to a level that allowed local surgical resection. The overall tumor progression was controlled to some extent, but a dramatic reduction could not be achieved. Bleeding from the previously hemorrhagic tumor was reduced to a certain extent, preventing further progression of anemia. The resection was performed with a 2 cm margin along the fascia, including part of the muscle tissue directly beneath the tumor.

**Figure 3:**
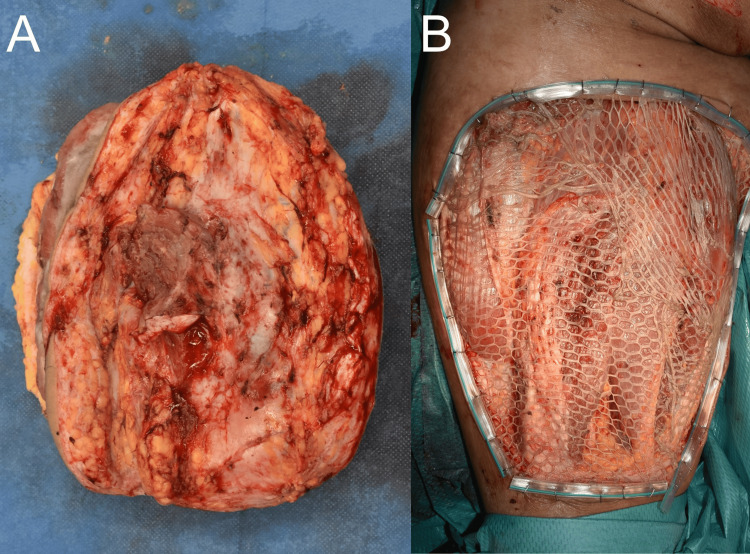
Clinical images (A) Tumor resection revealed partial infiltration into the underlying muscle. (B) After the resection, a split-thickness skin graft was performed.

The primary tumor was excised, and a layered skin graft with a 3× mesh was applied. Histological analysis revealed that the tumor had no continuity with the epidermis (Figures 3A, 3B).

**Figure 4:**
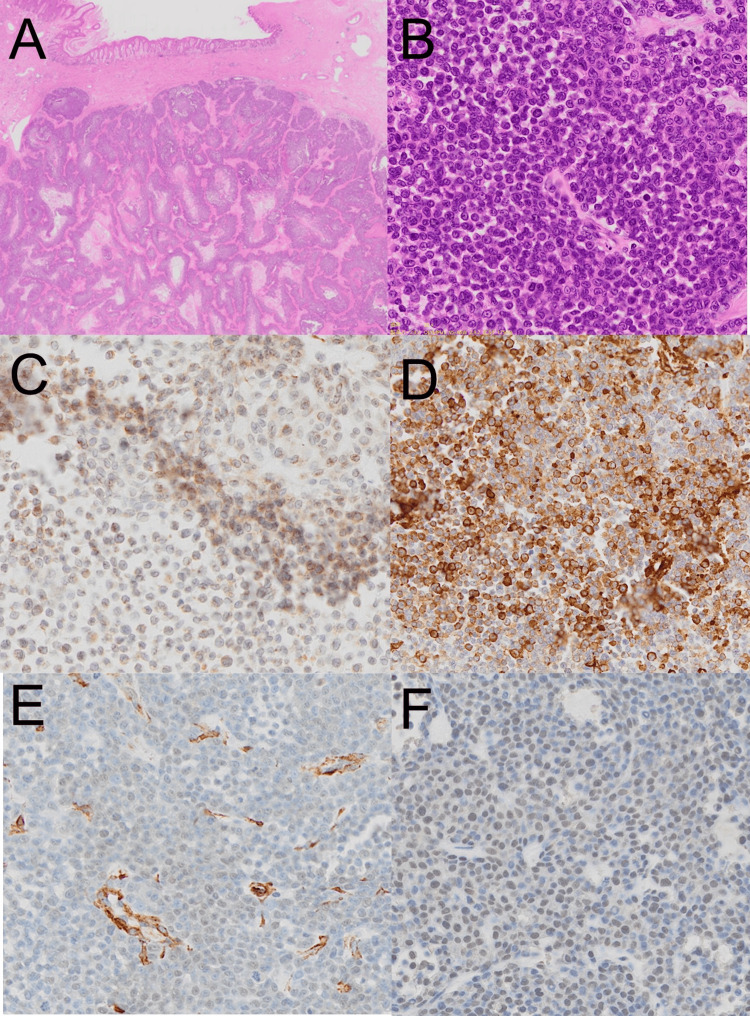
Histological and immunohistochemical findings (A) Histological slide showing the tumor with multiple, nodular, basophilic tumor nests infiltrating the dermis without a capsule; Magnification: x10. (B) High magnification view of the tumor cells exhibiting marked nuclear atypia with prominent nucleoli and numerous mitotic figures; Magnification: x100. (C) Immunohistochemical staining for calponin. (D) Immunohistochemical staining for α-SMA. (E) Immunohistochemical staining for HHF-35. (F) Immunohistochemical staining for PLAG1.

The tumor showed non-encapsulated, multiple, nodular, basophilic tumor nests infiltrating the dermis (Figures 4A, 4B). The tumor cells exhibited marked nuclear atypia with prominent nucleoli and numerous mitotic figures. The stroma showed dilated capillaries and infiltration by lymphocytes and spindle cells. Immunohistochemistry revealed partial positivity for calponin, α-SMA, HHF-35, and pleomorphic adenoma gene 1 (PLAG1) (Figures 4C-4F), indicating the diagnosis of primary cutaneous myoepithelial carcinoma.

After the palliative resection, the subsequent treatment included radiotherapy to both the metastatic lymph nodes in the left groin and the primary lesion. Additionally, tegafur gimeracil oteracil potassium was administered as part of the ongoing treatment regimen, resulting in the control of the disease without tumor progression for two years post-resection.

## Discussion

This case of a giant myoepithelial carcinoma, initially suspected to be SCC, underscores the diagnostic challenges posed by this rare tumor [1]. Accurate diagnosis necessitates immunohistochemical staining for specific myoepithelial markers such as α-SMA, HHF-35, and calponin [7]. Detailed observation of the tumor's growth pattern and histological characteristics is paramount. Myoepithelial carcinoma typically lacks a capsule and exhibits a multinodular infiltrative pattern with a mucinous or hyaline stroma [1]. A comprehensive pathological examination based on these features is essential for precise diagnosis.

In typical clinical presentations, myoepithelial carcinoma appears as a solid nodule [8]. In this particular case, the tumor likely underwent self-destruction and ulceration of the surface, leading to a clinical appearance resembling SCC [9]. The ulceration of the tumor surface may have masked its true nature, contributing to the initial manifestation of SCC in this case.

Thorough pathological evaluation, taking into account the tumor's unique characteristics and growth patterns, is crucial for the correct diagnosis. This case highlights the importance of considering myoepithelial carcinoma in the differential diagnosis of ulcerative tumors, especially when initial clinical and histological findings suggest SCC, basal cell carcinoma, Merkel cell carcinoma, and porocarcinoma, leading to appropriate therapeutic strategies based on a comprehensive understanding of the tumor's pathology.

Currently, there is no standardized chemotherapy regimen specifically established for myoepithelial carcinoma [10,11]. Indeed, platinum agents have the potential to effectively reduce tumors in cases of myoepithelial carcinoma [12]. However, more cases need to be documented and studied to establish detailed and reliable treatment protocols.

## Conclusions

In conclusion, this case of giant primary cutaneous myoepithelial carcinoma, initially misdiagnosed as SCC, underscores the critical importance of thorough pathological and immunohistochemical evaluation in cases of atypical ulcerative tumors. The rarity and unusual presentation of primary cutaneous myoepithelial carcinoma make it a diagnostic challenge, often leading to delays in establishing the correct diagnosis and initial misclassification. However, as demonstrated in this case, prompt diagnosis can significantly impact treatment decisions and outcomes. This case adds to the growing body of evidence supporting the need for heightened awareness and consideration of myoepithelial carcinoma in differential diagnoses, as well as highlights the value of innovative treatment strategies in improving patient prognosis.
